# Ageing, technology, and health: Advancing the concepts of autonomy and independence

**DOI:** 10.1177/08404704221110734

**Published:** 2022-08-04

**Authors:** Lili Liu, Christine Daum, Antonio Miguel Cruz, Noelannah Neubauer, Hector Perez, Adriana Ríos Rincón

**Affiliations:** 1 8430 University of Waterloo, Waterloo, Ontario, Canada.; 2 3158 University of Alberta, Edmonton, Alberta, Canada.; 3 Glenrose Rehabilitation Hospital, Edmonton, Alberta, Canada.

## Abstract

The global pandemic expedited the adoption of AgeTech solutions that aim to help older adults maintain their autonomy and independence. This article examines the negative impact of the Western worldview of autonomy and independence on older adults. Negative impact can manifest as ageism and may be compounded by intersections of identities with race, gender, and culture. We propose an inclusive framework for health leaders, one that is not binary or categorical, but instead, on a continuum: (1) relational autonomy which assumes that relationships form one’s identity; therefore, no one is autonomous to the exclusion of others, and (2) interdependence which proposes that one’s lifestyle choice is supported by interreliance with aspects of one’s environment. We examine two examples of AgeTech from the perspective of relational autonomy and interdependence and discuss how health leaders can use this inclusive framework to ensure that their services do not discriminate against older adults.

## Introduction

COVID-19 has accelerated the adoption of technologies that connect us in our personal lives, healthcare delivery, school, and work. These modalities of connecting people and services are here to stay after the pandemic.^1,2^ The adoption of technologies in older adult populations can support autonomy and independence. Generally, these technologies have been in the category of Information Communication Technologies (ICTs) that enable communication or access to services on-line.

AgeTech, also known as gerontechnology, is a category of technologies targeted to the needs of older adults.^3^ AgeTech includes ICT, robotics, mobile technologies, artificial intelligence, ambient systems, and pervasive computing to drive technology-based innovation to benefit older adults.^4^ More than ever, everyday digital technologies adopted by the mainstream population also benefit older adults. For example, smartphones provide numerous functions that people now rely on, including navigation, video calls, gaming, reading the news, listening to music, watching videos, and social media.

However, segments of the population do not have easy access to technologies. This digital divide is evident among recent immigrants, individuals with lower income and education, and those living in rural communities.^5^ According to the Canadian Radio-television and Telecommunications Commission, 89.5% of Canadian households have access to broadband internet, but only 53.4% of rural communities do.^6^ Access to technologies by people who reside in rural areas can improve with policies that increase infrastructure and make them affordable.

Despite the rapid adoption and diffusion of technologies among health services at the onset of the pandemic, there is still a digital divide for older adults.^2,3^ However, this gap is narrowing. Over a third used social media to communicate with friends and family, and 19% used on-line shopping for groceries and “health, wellness, or independence” at the onset of the pandemic.^7^ According to the Pew Research Centre in the United States,^8^ adults aged 65 and older lag behind younger adults, aged 18 to 29, in owning a smartphone (61% vs. 96%), using social media (45% vs. 84%), and owning a tablet computer (44% vs. 46%), but the difference has reduced over the past decade.

A purpose of AgeTech is to enable autonomy and independence in older adults. However, a tendency to use the terms autonomy and independence interchangeably may subject older adults to unintentional ageism. We begin by examining these terms.

## Autonomy

Traditionally, autonomy refers to an ability to make decisions for oneself and equates to responsibility, integrity, dignity, individuality, and self-knowledge.^9,10^ Autonomy is one of the fundamental ethical principles of healthcare,^11^ and a human right protected by universal conventions and declarations.^12,13^ Autonomy can be examined at the level of an individual and also at the level of a community. At the individual level, older adults are respected for their ability to make decisions for themselves. This ranges from basic decisions about food choices to more complex decisions related to health, such as whether to undergo surgery. At the community level, autonomy refers to the right of a group of individuals to determine their choices and actions collectively. According to Hall,^14^ this is an extension of individual autonomy.

## Independence

Typically, independence is prioritized over autonomy. Independence is generally the focus and goal of health services, particularly rehabilitation. Independence is conceptualized as a person’s ability to perform an activity or to “do things” for oneself without help from someone else^15^ and is viewed as a marker of health. Independence is associated with successful ageing and ageing well. This can be observed in the plethora of assessment tools that evaluate an older adult’s physical and cognitive ability to perform basic activities of daily living (bathing, dressing, and feeding), mobility (walking or ambulating), and instrumental activities of daily living (navigating the community, looking after the home, finances, and managing medication). Common screening and assessment tools that focus on independence include the Barthel Index,^16^ the Functional Independence Measure,^17,18^ and the Assessment of Motor and Process Skills.^19,20^ In Western culture, individualism, self-reliance, and competence are emphasized.^21^ Independence is closely aligned with living independently and is highly valued. And, for some who hold the Western worldview, independence becomes a “moral imperative”;^22^ dependence is considered undesirable.^23^

## Advancing the concepts of autonomy and independence

Clearly, autonomy and independence are not interchangeable concepts. An individual may be dependent on services and technology for self-care tasks yet feels a sense of autonomy through an ability to make meaningful choices. Autonomy refers to choice and may remain intact even for an individual dependent on others for self-care activities. Conversely, a person may be independent in activities yet experience a lack of autonomy regarding decisions due to the social, physical or political environment that impedes meaningful engagement. Meaningful activities contribute to an older adult’s sense of identity.^24^ When autonomy is expressed in the form of engagement in meaningful activities, there is a positive impact on one’s psychosocial and physical well-being.^24,25^

People’s interests, needs, identity, and, inevitably, their autonomy are shaped by their relation to others.^26^ It is within these relationships and social conditions that one’s autonomy emerges.^27^ When respected, autonomy can be recognized regardless of the place of residence and level of capacity of an older adult. An older adult living with late-stage dementia can express preferences and disinterest through non-verbal communication, facial expressions, and gestures.

Individualism and independence are emphasized and desired particularly in the Western worldview but may not be valued by all cultures.^28^ In East Asian and Indigenous worldviews, collectivism, rather than individualism, is dominant. People are perceived as inextricably connected and entwined with the land and people.^23^ Dependence on others is not viewed negatively or burdensome. Instead, a degree of dependence on others represents being connected.

In the West, independence is typically measured as an ability to do basic and instrumental activities of daily living *without* help from others or devices such as a mobility aid or assistive technology. In reality, independence is an illusion. As Portacolone^22^ aptly states, “…we live in a constant and often invisible interdependence with one another—with institutions, family, friends, strangers, and adult day centres.” We do not live in isolation from the natural environment, the people, and the objects within it. If we use supports, does that mean we are partially dependent or partially independent? In the context of older adults, if a person lives in a private home in the community with cleaning, driving, and meals services and assistive technologies to make the home safe, a companion who provides social visits, and a network of family and friends coordinate these services, again, do we consider the person to be partially dependent and or partially independent? In contrast, do we consider an older adult who lives in a supportive living facility in which she receives housekeeping services and one meal a day and uses public transit to venture into the community to participate in activities, to be less independent than another older adult who lives in the community without the supportive services? Where does independence end and dependence begin?

The current view of independence also does not consider people’s choices, histories, and priorities. For example, when faced with chronic conditions that impact endurance, a person may choose to prioritize activities that are most meaningful or enjoyable. The person may consciously *choose* not to do some activities (eg, laundry and cleaning). Yet, this person may be labelled as being dependent although the person is exercising autonomy.

## Relational autonomy and interdependence

Relational autonomy expands the concept of autonomy by incorporating aspects that support one’s choices.^3,29^ These include aspects of an individual’s identity, family, social network, and spirituality. Nedelsky^29^ posits that as unique individuals, our identities are formed through the social interactions and relationships we develop. It is understandable and should be expected that when individuals make choices about their healthcare, they consult with or refer to their experiences and relationships, such as family and social networks, spiritual beliefs, and persons they consider to be experts. This approach is inclusive of cultures beyond the Western worldview. For example, “Two-Eyed Seeing” is an approach coined by Bartlett et al.^30^ to describe the integration of traditional Indigenous and mainstream sciences and ways of knowing. However, it is common that when a person receives a diagnosis of a chronic condition such as dementia, the person’s autonomy is not respected due to stigma and an assumption that the person’s ability to make choices is impaired.^31^

**Figure 1.**
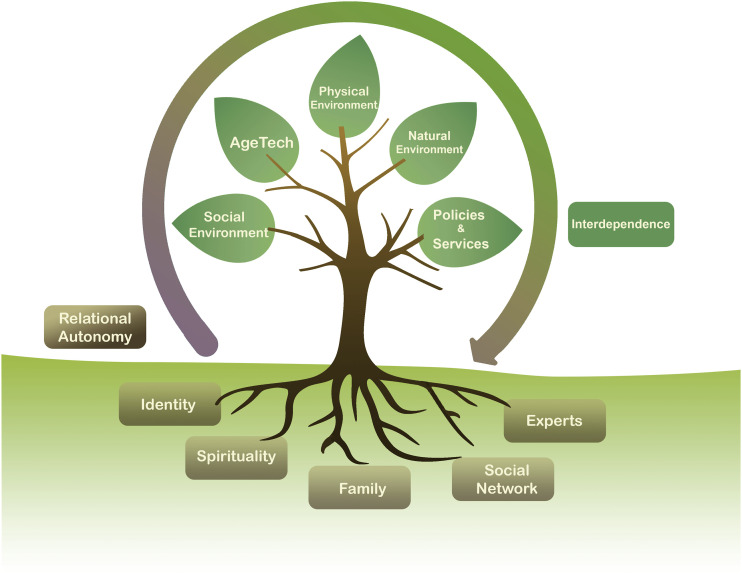
Framework for relational autonomy and interdependence.

If we consider aspects of relational autonomy to be the roots in Figure 1, forming the foundation that captures elements that inform a person’s choices, the branches represent the analogous concept for independence, which we call interdependence just as “nothing can ever be only one’s own.”^29^ Nedelsky^29^ rejects that an individual is entirely independent; rather, independence can be described as the “less dependent” end of a spectrum.^32^

Individuals can be interdependent on various aspects of their lives. These include social environment or connections, AgeTech, one’s physical or built environment, the natural environments, and policies and services as depicted in the branches of Figure 1.

Interdependence acknowledges the interconnection between people and the “human and non-human” aspects of their lives.^33^ As Fine and Glendinning^34^ point out, recognizing interdependence “is not to deny but to acknowledge relations of dependence.” In other words, dependence is a typical feature of life, a reality in caring relationships, and a component of interdependence. Interdependence suggests that there is reciprocity between people.^34^ The exchanges that occur when people are interdependent may not be immediate but build over time. For example, interdependence occurs in co-housing situations where people live in their own suites but share responsibilities for home maintenance, meal planning, and social engagement.^35^ Interdependence also occurs in naturally occurring retirement communities.^36^ Likewise, it occurs when older couples work together as one unit so that they may successfully perform their daily activities following a change in health status.^37^ In some cultures, interdependence is the norm of everyday family life; when a member of a family or social circle requires care, other members of the circle pitch in to support the person in need. Interdependence not only refers to dependencies on people but also on systems, objects, and technologies or AgeTech. A study that examined how older adults with mobility restrictions organize their out-of-home mobility and independent living revealed that mobility was the result of interdependence, not only a person’s capabilities and self-sufficiency.^33^ Independence and interdependence are not mutually exclusive; as Portacolone^22^ states, “Independence and interdependence are complementary: one can feel a sense of independence while being connected with others.”

In healthcare, when service providers focus on “autonomy” and “independence” in assessments and interventions, the complex nature of individuals’ background, social connections, culture, and motivations become reduced to a checklist of performance capacity. The language in outcome measures directs us to focus on dichotomous ratings of dependence versus independence, with little regard for the impact of these assessments on our clients’ sense of autonomy in the expression of choices. The quote “safety is what we want for those we love, and autonomy is what we want for ourselves”^38^ illustrates paternalism in healthcare at the expense of autonomy. In this way, older adults can experience ageism in healthcare.

## Interdependence on AgeTech

As depicted in Figure 1, interdependence on one’s social, physical, and natural environments, along with policies and services, and AgeTech can facilitate one’s choices to live in a certain way. Ubiquitous technologies such as smartphones and tablets and the internet have made it possible for populations to connect socially during periods of COVID-19 pandemic lockdown.

Interdependence on AgeTech can enhance the quality of life for older adults. In the book Autonomy and Independence: Ageing in an era of technology,^3^ we provide 11 personas and numerous examples of AgeTech that facilitate self-care, management of one’s health, mobility, and caregiving, among others. Below we provide two examples.

The first example of a common type of AgeTech is serious digital games. While digital games are familiar to players across the lifespan, a “serious” game is one that is used for therapeutic purposes.^39^ Healthcare practitioners recognize the benefits and opportunities of these platforms. Some older adults find interactive digital games more engaging compared to traditional games. Therapists appreciate the unobtrusive way these games can collect data on performance. As the tablet or digital platform is ubiquitous, it carries less stigma. Interactive digital games can potentially evaluate a broader spectrum of cognitive abilities than traditional approaches using questionnaires. For example, using a “Whack-a-Mole” digital game on a tablet, a person with advanced dementia demonstrated improvement in game-playing over 10 weeks despite declining mental status performance.^40^ In this example, the person’s behaviour was “less dependent” when engaged with the digital game, compared to her scores on the Mini-Mental Status Examination, which were consistent with progressively “more dependent.”

The second example is social robots. Several types of robots exist as everyday technologies, including robot vacuums that can be pre-programmed to automatically clean an area of the floor in one’s home. These are different from robots that assist human users with specific functions, such as picking up and moving items to compensate for a person’s impaired function.^41^ That said, assistive robots like Stretch^42^ can promote relational autonomy by serving as an extension of the user to perform gestures that strengthen bonds of affection, such as giving a flower to his spouse, in the case of Henry Evans, a man living with quadriplegia. Stretch can perform household chores like vacuuming, playing fetch with the dog and help Evans with self-care activities such as shaving.^42^ This provides Evans with a sense of autonomy and self-worth. At $20,000, this type of robot is becoming affordable for some and can be realistically placed in homes.

## Rediscovering autonomy and independence in the Canadian healthcare system

By conceptualizing autonomy and independence as separate concepts on continuums of relational autonomy and interdependence, we can shift our mindset toward these terms in our healthcare system. Self-managed care is enabled through provincial on-line platforms allowing clients and their care partners to access lab test results, such as in Alberta and Ontario. Advance care planning and autonomy can be facilitated by incorporating technologies such as digital applications linked to a client’s health records.^43^ Such care planning can include wishes and instructions for future use of AgeTech, including monitoring devices. Digital storytelling, the use of digital media to record life events, can support autonomy and self-determination, helping to share one’s choices and legacy with loved ones before cognitive decline and dependence set it.^44^ A digital story can remind care partners and staff of patients’ preferences when they can no longer communicate, thus preserving their autonomy. Self-managed care, advanced care planning, and digital story telling are examples of how one’s autonomy can be respected even when a person becomes dependent on others for self-care. Ageism occurs when we are not aware of nor respect the autonomy of an older adult who is dependent on help.

## Conclusion

A Western worldview dominates autonomy and independence. This is associated with a dichotomous perspective of capacity in older adults, putting them at risk for ageism. We propose an inclusive framework that describes relational autonomy as a set of aspects that inform a person’s choices regarding daily activities. After a health service provider has considered a person’s relational autonomy, assessments and interventions are viewed from the perspective of interdependence. Supportive social, physical, and natural environments and access to policies and services can enable older adults’ choices. In the case of AgeTech, an older adult’s need for or reliance on technology is viewed as a strategy to enhance a person’s quality of life, not as a limitation in capacity.
